# The mycobiome of Australian tree hollows in relation to the *Cryptococcus gattii* and *C. neoformans* species complexes

**DOI:** 10.1002/ece3.5498

**Published:** 2019-08-07

**Authors:** Laura J. Schmertmann, Laszlo Irinyi, Richard Malik, Jeff R. Powell, Wieland Meyer, Mark B. Krockenberger

**Affiliations:** ^1^ Sydney School of Veterinary Science The University of Sydney Sydney NSW Australia; ^2^ Molecular Mycology Research Laboratory, Centre for Infectious Diseases and Microbiology, Faculty of Medicine and Health, Westmead Clinical School The University of Sydney Sydney NSW Australia; ^3^ The Westmead Institute for Medical Research Westmead NSW Australia; ^4^ Marie Bashir Institute for Infectious Diseases and Biosecurity The University of Sydney Sydney NSW Australia; ^5^ Centre for Veterinary Education, Sydney School of Veterinary Science The University of Sydney Sydney NSW Australia; ^6^ Hawkesbury Institute for the Environment Western Sydney University Penrith NSW Australia

**Keywords:** Australia, cryptococcosis, *Cryptococcus*; mycobiome, eucalypt, tree hollow

## Abstract

Cryptococcosis is a fungal infection caused by members of the *Cryptococcus gattii* and *C. neoformans* species complexes. The *C. gattii* species complex has a strong environmental association with eucalypt hollows (particularly *Eucalyptus camaldulensis*), which may present a source of infection. It remains unclear whether a specific mycobiome is required to support its environmental survival and growth. Conventional detection of environmental *Cryptococcus* spp. involves culture on differential media, such as *Guizotia abyssinica* seed agar. Next‐generation sequencing (NGS)‐based culture‐independent identification aids in contextualising these species in the environmental mycobiome. Samples from 23 Australian tree hollows were subjected to both culture‐ and amplicon‐based metagenomic analysis to characterize the mycobiome and assess relationships between *Cryptococcus* spp. and other fungal taxa. The most abundant genera detected were *Coniochaeta*, *Aspergillus*, and *Penicillium*, all being commonly isolated from decaying wood. There was no correlation between the presence of *Cryptococcus* spp. in a tree hollow and the presence of any other fungal genus. Some differences in the abundance of numerous taxa were noted in a differential heat tree comparing samples with or without *Cryptococcus*‐NGS reads. The study expanded the known environmental niche of the *C. gattii* and *C. neoformans* species complexes in Australia with detections from a further five tree species. Discrepancies between the detection of *Cryptococcus* spp. using culture or NGS suggest that neither is superior per se and that, rather, these methodologies are complementary. The inherent biases of amplicon‐based metagenomics require cautious interpretation of data through consideration of its biological relevance.

## 1 INTRODUCTION

Cryptococcosis is a potentially lethal mycosis affecting both humans and animals, caused by basidiomycetous yeasts in the *Cryptococcus gattii* and *C. neoformans* species complexes. Molecular typing based on, for example, PCR‐fingerprinting (Meyer & Mitchell, 1995; Viviani et al., 1997), AFLP analysis (Boekhout et al., 2001), *URA5‐*RFLP (Meyer, Castañeda, Jackson, Huynh, & Castañeda, 2003) analysis, and MLST studies (Meyer et al., 2009) have further divided these species complexes into major molecular types, with *C. gattii* comprising the major molecular types VGI = AFLP4, VGII = AFLP6, VGIII = AFLP5, and VGIV = AFLP7 and *C. neoformans* comprising the major molecular types VNI = AFLP1, VNII = AFLP1B, VNB = AFLP1A, the AD hybrid VNIII = AFLP3 and VNIV = AFLP2 (Boekhout et al., 2001; Meyer et al., 2009). Recently, it was suggested that some of these major molecular types should be raised to species level (Hagen et al., 2015). However, the proposed division of these complexes into seven distinct species is still the subject of debate (Hagen et al., 2017; Kwon‐Chung et al., 2017) and a recent publication has shown that this proposal is premature (Cogliati et al., 2019). To maintain taxonomic stability, the use of “species complexes” had been proposed as interim solution (Kwon‐Chung et al., 2017) and is used herein.

Cryptococcosis is acquired from the environment by the inhalation of basidiospores or desiccated yeast cells (Kwon‐Chung et al., 2014). The *C. gattii* and *C. neoformans* species complexes inhabit various ecological niches and are associated with decaying organic material, such as wood, soil and pigeon excreta (Cogliati et al., 2016; Kidd et al., 2007; Nielsen, De Obaldia, & Heitman, 2007; Springer et al., 2014). The ecological role of *Cryptococcus* spp. is not well understood but their presence in decaying organic matter suggests that they might contribute to the process of decomposition (Voříšková & Baldrian, 2013).

The *C. gattii* species complex has traditionally been regarded as tropical or subtropical, whereas the *C. neoformans* species complex is globally distributed. There is increasing evidence, however, that the *C. gattii* species complex is also prevalent in temperate climates (Chowdhary et al., 2012; Colom et al., 2012; Kidd et al., 2004). Ellis & Pfeiffer described a specific ecological association between the *C. gattii* species complex and eucalypt trees in Australia, most notably the river red gum (*Eucalyptus camaldulensis*) (Ellis & Pfeiffer, 1990), in a wide variety of temperate and subtropical locations. Since then, the *C. gattii* species complex has been found globally in decaying wood, particularly inside trunk hollows and in various living tree species, suggesting that trees could be its primary natural habitat (Cogliati et al., 2016; Kidd et al., 2007; Lazera et al., 2000; Randhawa et al., 2008).

In Australia, eucalypts appear to be a key environmental niche for the *C. gattii* species complex (particularly *C. gattii* VGI), and the range of tree species from which it has been isolated continues to expand (Table 1). Koalas (*Phascolarctos cinereus*) exhibit comparatively high rates of both clinical and subclinical cryptococcosis, likely preceded by nasal colonization by *Cryptococcus* spp., with this association presumed to be related to their close association with eucalypts (Krockenberger, Canfield, & Malik, 2003). In recent years, several cases of cryptococcosis have been observed in free‐ranging koalas (*Phascolarctos cinereus*) inhabiting the Port Stephens (Schmertmann et al., 2018) and Liverpool Plains (Schmertmann et al., 2019) regions of New South Wales, Australia. As the Vancouver Island *C. gattii* VGII outbreak highlighted the potential for animals to represent key sentinels for human disease (Lester et al., 2004), we were prompted to conduct environmental investigations in both areas.

**Table 1:** Tree species from which the *Cryptococcus gattii* species complex has been isolated in Australia, including previously published data

Scientific name	Common name	*Cryptococcus* spp. association	Method of first detection	Source of first detection
*Angophora costata*	Smooth‐barked apple	*C. gattii* VGI	Culture	Halliday et al. (1999)
*Eucalyptus albens*	White box	*C. gattii* VGI	Culture	Schmertmann et al. (2019)
*E. bella*	Ghost gum	*C. gattii* VGII	Culture	Sorrell et al. (1996)
*E. blakelyi*	Blakely's red gum	*C. gattii* VGI	Culture	Pfeiffer and Ellis (1996)
*E. camaldulensis*	River red gum	*C. gattii* VGI	Culture	Ellis and Pfeiffer (1990)
*E. gomphocephala*	Tuart	*C. gattii* VGI	Culture	Pfeiffer and Ellis (1996)
*E. grandis*	Flooded gum	*C. gattii* VGI	Culture	Halliday et al. (1999)
*E. microcorys*	Tallowwood	*C. gattii* VGI	Culture	Krockenberger, Canfield, and Malik (2002)
*E. rudis*	Flooded gum	*C. gattii* VGI	Culture	Pfeiffer and Ellis (1996)
*E. tereticornis*	Forest red gum	*C. gattii* VGI	Culture	Pfeiffer and Ellis (1992)
*Syncarpia glomulifera*	Turpentine gum	*C. gattii* VGI	Culture	Krockenberger et al. (2002)
***A. floribunda***	**Rough‐barked apple**	***C. gattii* VGI, *C. neoformans* VNI/VNII**	**Culture, NGS**	**This study**
***E. pilularis***	**Blackbutt**	***C. gattii* VGI, *C. neoformans* VNI/VNII**	**Culture, NGS**	**This study**
***E. populnea***	**Poplar box**	***C. gattii* VGI**	**NGS**	**This study**
***E. robusta***	**Swamp mahogany**	***C. gattii* VGI**	**Culture**	**This study**
***Melaleuca* spp.**	**Paperbark**	***C. gattii* VGI**	**Culture**	**This study**

Bold values indicate newly identified tree species hosting *Cryptococcus* spp.

Abbreviation: NGS, next‐generation sequencing.

There has been ongoing speculation regarding whether the presence of specific fungal communities or critical species support the growth of *Cryptococcus* spp. in the environment. A study conducted in Africa attempted to characterize this notion but did not find any associations between the presence of any specific fungal taxa and *Cryptococcus* spp. (Vanhove et al., 2017). The fungal community residing in eucalypt tree hollows in association with the *C. gattii* species complex in Australia remains unexplored.

The detection of *Cryptococcus* spp. from the environment is important in the context of cryptococcosis in order to pinpoint potential infection sources, with culture being the primary means of achieving this. Staib's niger (*Guizotia abyssinica*) seed extract agar containing antibiotics is a differential and selective media that underpins the conventional detection of *Cryptococcus* spp. from environmental samples using mycological culture (Paliwal & Randhawa, 1978; Shields & Ajello, 1966). The method is based on *Cryptococcus* spp. colonies exhibiting the brown‐color‐effect, due to the production of melanin by cryptococcal laccase (a phenoloxidase enzyme; Staib, 1962). This method is dependent on the visual recognition of suspected cryptococcal colony‐forming units (CFUs), which can be challenging when multiple fast‐growing filamentous fungi are presented concurrently.

New culture‐independent methods, such as next‐generation sequencing (NGS), have the potential to detect any organism, including *Cryptococcus* spp., in the environment without relying on growth in vitro, therefore aiding in the identification of sources of infections. In addition, they can also define the microbial communities present in environmental samples (Hamad et al., 2017; Taberlet, Coissac, Pompanon, Brochmann, & Willerslev, 2012; Tong et al., 2017). There has been a fundamental shift away from conventional DNA sequencing introduced by Sanger, Nicklen, and Coulson (1977), considered as first‐generation sequencing technology, to newer methods, such as NGS. High‐throughput sequencing technologies have revolutionized biological research and allowed for the in‐depth characterization of microbial diversity, without the need for morpho‐taxonomy (Creer et al., 2016).

Fungi might represent the largest genetic diversity among the eukaryotes, with an estimated 5.1 million species, including taxa ranging from unicellular yeasts and microscopic molds to large mushrooms (Blackwell, 2011). Of this enormous number of species, only a small number are known to be potential mammalian pathogens. To overcome inherent limitations of culture‐based methods of characterizing microbial communities, indirect molecular methods have been developed based on the total DNA content of the sample. Amplicon‐based metagenomics analyses (metabarcoding) have become a widely used technology in various fields, ranging from microbial ecology studies to infectious disease surveillance (Nguyen, Viscogliosi, & Delhaes, 2015; Tedersoo et al., 2014; Tonge, Pashley, & Gant, 2014). Currently, it is the standard tool and the most efficient method for culture‐independent assessment of microbiomes, even if its broad application is still hampered by relatively high cost and the need for special bioinformatic analyses (Tang, Iliev, Brown, Underhill, & Funari, 2015). The approach combines the methodologies of DNA barcoding (Hebert, Cywinska, Ball, & deWaard, 2003) with high‐throughput sequencing technology. It is based on the concept that each operational taxonomic unit (OTU) can be unequivocally identified using DNA barcodes. The general strategy involves the following: (a) extraction of DNA from an environmental sample or organism, (b) amplification of the species‐specific DNA barcodes, (c) sequencing of the DNA amplicons, (d) analyses of the generated sequences using appropriate pipelines, and (e) taxonomic assignment of the detected sequences. PCR‐based metabarcoding has become a rapid and accurate method to species level identification from complex environmental and clinical samples, without the requirement for culture, thereby providing unprecedented insights into the underlying biological diversity (Bik et al., 2012).

In this study, we used amplicon‐based NGS as a tool to characterize the fungal community (mycobiome) of tree hollows, allowing assessment of the coexistence of *Cryptococcus* spp. with other fungal genera in eucalypt and other Australian native tree hollows in two areas of New South Wales (NSW), Australia. The two areas have recently been associated with cryptococcosis in koalas. We also compared conventional culture‐based methods with NGS, to determine which was more sensitive at detecting pathogenic *Cryptococcus* spp. in environmental samples.

## 2 MATERIALS AND METHODS

### 2.1 Sample collection

Debris and related material from hollows were collected from 23 trees selected randomly at multiple locations within the Port Stephens (9) and Liverpool Plains (14) regions of New South Wales, Australia (Table 2). Tree species from which samples were taken included *E. camaldulensis* (12), *E. pilularis* (2), *E. tereticornis* (2), *Eucalyptus* spp. (2), *Angophora floribunda* (1), *E. albens* (1), *E. populnea* (1), *E. robusta* (1), and *Melaleuca* spp. (1). Samples were collected as part of a disease investigation into cases of koala cryptococcosis. Generous amounts of material were collected from the interior of each tree hollow and placed into clean plastic bags, which were sealed and labeled then maintained at room temperature.

**Table 2:** Detection of *Cryptococcus* spp. from 23 tree hollow samples in New South Wales, Australia by using conventional culturing and next‐generation sequencing

Sample	Tree species	Region	Culture/*URA5*‐RFLP results	Number of reads detected with amplicon‐based (ITS1) next‐generation sequencing		
			*Cryptococcus gattii* VGI	Total reads	*C. gattii* VGI	*C. neoformans* VNI/VNII
**E2699**	***Melaleuca* spp**.	**PS**	**+**	**152,490**	**–**	**–**
E2771	*Eucalyptus camaldulensis*	LP	+	149,970	2	–
E2666	*Angophora floribunda*	PS	++	125,784	3	1
**E2697**	***E. robusta***	**PS**	**++**	**89,683**	**–**	**–**
E2704	*E. tereticornis*	PS	++	226,974	1	1
E2768	*E. camaldulensis*	LP	++	70,250	1	1
E2772	*E. camaldulensis*	LP	++	206,302	11	10
E2773	*E. camaldulensis*	LP	++	152,861	3	7
E2774	*E. camaldulensis*	LP	++	129,923	2	2
**E2657**	***E. pilularis***	**PS**	**+++**	**131,034**	**–**	**–**
E2760	*E. albens*	LP	+++	104,095	6	2
E2761	*E. camaldulensis*	LP	+++	104,725	52	1
E2764	*E. camaldulensis*	LP	+++	142,580	4	4
E2770	*E. camaldulensis*	LP	+++	145,686	11	5
E2668	*E. tereticornis*	PS	−	158,773	–	–
E2677	*Eucalyptus* spp.	PS	−	114,688	–	–
E2698	*Eucalyptus* spp.	PS	−	126,651	–	–
E2711	*E. pilularis*	PS	−	71,419	–	–
**E2757**	***E. populnea***	**LP**	**–**	**105,099**	**3**	**–**
**E2762**	***E. camaldulensis***	**LP**	**–**	**133,183**	**5**	**–**
**E2765**	***E. camaldulensis***	**LP**	**–**	**118,666**	**1**	**–**
**E2766**	***E. camaldulensis***	**LP**	**−**	**163,204**	**1**	**1**
**E2769**	***E. camaldulensis***	**LP**	**−**	**108,789**	**6**	**7**

Note

Samples are ordered by positive or negative culture results and degree of growth. Bold text denotes samples in which culture and next‐generation sequencing results were discordant.

Abbreviations: LP = Liverpool Plains region, New South Wales, Australia; PS = Port Stephens region, New South Wales, Australia; RFLP = restriction fragment length polymorphism; + = Low (1–10 cryptococcal colony‐forming units [CFUs] on culture), ++ = moderate (11–100 CFUs), +++ = heavy (>100 CFUs).

### 2.2 Culture

Samples were inoculated onto niger seed extract agar as soon as possible by introducing a sterile swab, premoistened with sterile saline, into the sample and then gently rolling the swab across the agar plate. Plates were incubated at 27°C for a minimum of 7 days and monitored daily. *Cryptococcus* spp. CFUs were identified by the brown‐color‐effect and their yeast‐like growth on niger seed agar. Once suspected cryptococcal CFUs were observed, the agar plate was removed from the incubator and one or more CFUs were subcultured onto Sabouraud's dextrose agar for isolation of a pure culture, which was followed by DNA extraction (see below). Samples E2657, E2666, and E2704 had five, four and three isolates collected, respectively, from each primary isolation plate. In all other positive samples, only one isolate was collected. DNA extraction from cryptococcal isolates was performed using an adaptation of an established fungal DNA extraction method (Ferrer et al., 2001). Restriction fragment length polymorphism (RFLP) analysis of a *URA5* PCR product was used as described previously (Meyer et al., 2003) to determine the cryptococcal species and molecular type of each isolate.

### 2.3 DNA Extraction for NGS

DNA was extracted directly from tree hollow material by initially grinding approximately 20 g of each sample with liquid nitrogen using a mortar and pestle. This homogenized the sample and aided in breaking down both the cryptococcal capsule and fungal cell walls. DNA was then extracted from the ground portion of the sample using the DNeasy PowerSoil kit (Qiagen GmbH) following manufacturer's instructions.

### 2.4 PCR amplification of the ITS1 region

Fragments were amplified with the universal fungal primers, ITS1F (CTTGGTCATTTAGAGGAAGTAA) and ITS2 (GCTGCGTTCTTCATCGATGC) (White, Bruns, Lee, & Taylor, 1990) targeting the ITS1 region of the rRNA gene. PCR amplicons were generated using AmpliTaq Gold 360 Master Mix (ThermoFisher Scientific, North Ryde, NSW, Australia) for the primary PCR with the following amplification protocol: 7 min initial denaturing at 95°C, followed by 35 cycles of 30 s at 94°C, 45 s annealing at 55°C, 60 s at 72°C and 7 min final extension at 72°C. A secondary PCR to index the amplicons was performed with TaKaRa Taq DNA Polymerase (Clontech Laboratories).

### 2.5 Sequencing

Sequencing of PCR amplicons was conducted with MiSeq^®^ System of Illumina (Illumina) by the Australian Genome Research Facility. The Illumina bcl2fastq 2.18.0.12 pipeline was used to generate the sequence data. Pair‐ends reads 2 × 300 bp were generated up to 0.15 GB per sample.

### 2.6 Bioinformatics pipeline and analysis

Reads were processed according to the protocol described in the USEARCH documentation (https://drive5.com/usearch/manual/uparse_pipeline.html) using USEARCH package (Edgar, 2010) version 10.0. The sets of OTU were generated, “zero‐radius OTUs” (ZOTUs) (i.e., error‐corrected (denoised) sequences), using the UNOISE algorithm including chimera filtering (Edgar, 2016b) to identify all correct biological sequences in the reads. The ZOTU table was normalized to the same number of reads per sample (5,000) prior to downstream analysis, to be able to compare data from different measurements. All singletons were kept for downstream analysis. The taxonomy was predicted for ZOTU sequences using the SINTAX classifier (Edgar, 2016a) against the most recent combined (available on 04.03.2017) UNITE full dataset (Kõljalg et al., 2013) and ISHAM‐ITS (Irinyi et al., 2015) database containing all relevant ITS sequences of *Cryptococcus* spp. The SINTAX algorithm predicts taxonomy by using k‐mer similarity to identify the top hit in a reference database, supported by bootstrap values for all ranks in the prediction (Edgar, 2016a). All identified OTUs were retained for downstream analysis, including singletons.

The normalized abundances of OTUs for each sample were used to determine Shannon and Evenness indices as indicators of soil microbial diversity and structure, respectively. α‐diversity metrics were calculated using USEARCH package (Edgar, 2010) version 10.0 (Table 3). To estimate the potential correlation between *Cryptococcus* spp. and other genera, Pearson and Spearman co‐occurrence coefficients were then calculated with SparCC (Friedman & Alm, 2012) for each sample, separately. Taxon pairs with SparCC values >0.6 were considered as exhibiting a co‐occurrence relationship with a positive correlation.

**Table 3:** Estimations of fungal species richness of 23 tree hollows in New South Wales, Australia obtained with different models

Sample	Number of raw reads	Diversity metrics			Evenness metrics			
		Richness	Shannon_2	Jost1	Simpson	Dominance	Berger_Parker	Robbins
E2657	131,034	245	3.41	10.6	0.195	0.805	0.378	0.187
E2666	125,784	535	4.1	17.1	0.158	0.842	0.342	0.185
E2668	158,773	482	3.65	12.5	0.284	0.716	0.522	0.197
E2677	114,688	323	3.44	10.8	0.192	0.808	0.312	0.29
E2697	89,683	125	0.265	1.2	0.952	0.0478	0.976	0.413
E2698	126,651	143	2.96	7.79	0.222	0.778	0.342	0.319
E2699	152,490	524	4.1	17.2	0.188	0.812	0.367	0.145
E2704	226,974	520	5.84	57.4	0.0381	0.962	0.0938	0.0768
E2711	71,419	216	3	7.99	0.213	0.787	0.376	0.313
E2757	105,099	940	6.89	118.5	0.0222	0.978	0.0726	0.119
E2760	104,095	803	6.2	73.6	0.051	0.949	0.192	0.149
E2761	104,725	780	5.17	36	0.0853	0.915	0.244	0.25
E2762	133,183	537	3.37	10.3	0.217	0.783	0.405	0.229
E2764	142,580	461	4.06	16.7	0.194	0.806	0.416	0.24
E2765	118,666	560	3.7	13	0.214	0.786	0.427	0.271
E2766	163,204	1,062	6.41	85.1	0.0343	0.966	0.113	0.148
E2768	70,250	780	5.61	48.7	0.102	0.898	0.304	0.213
E2769	108,789	776	4.46	22	0.212	0.788	0.45	0.227
E2770	145,686	701	5.78	55.1	0.051	0.949	0.175	0.172
E2771	149,970	894	5.73	52.9	0.0665	0.933	0.163	0.169
E2772	206,302	228	4.63	24.7	0.0811	0.919	0.177	0.17
E2773	152,861	683	4.82	28.2	0.0997	0.9	0.246	0.161
E2774	129,923	808	4.36	20.5	0.237	0.763	0.477	0.193

Note

The diversity and evenness metrics were calculated on a normalized read number (5,000).

Richness: Number of operational taxonomic units (OTUs) with at least one read for the sample.

Shannon_2: Shannon index logs to base 2. It accounts for both abundance and evenness of the species present in the sample.

Jost1: Jost index of order 1, the effective number of species given by the Shannon index.

Simpson: the probability that two randomly selected reads will belong to the same OTU. A value close to 1 indicates that a single large OTU dominates the sample, small values indicate that the reads are distributed over many OTUs.

Dominance: Probability that two randomly selected reads will belong to different OTUs.

Berger_Parker: Frequency of the most abundant OTU. A value close to 1 indicates that a single large OTU dominates the sample, small values indicate that the reads are distributed over many OTUs.

Robbins: Robbins index, calculated as *S*/(*N* + 1) where *S* is the number of singleton OTUs and *N* is the total number of OTUs.

MetacodeR was used to visualize the mycobiome diversity of tree hollows in heat tree format (Foster, Sharpton, & Grünwald, 2017; R Core Team, 2013). A differential heat tree was created to indicate which taxa are more abundant in the presence of any *Cryptococcus* spp. reads detected by NGS. For statistical support, one‐sample Wilcoxon signed‐rank test was used in R (R Core Team, 2013) to test the hypothesis that certain taxa may be enriched for lower *p* value ranks than other taxa. Benjamini‐Hochberg (FDR) correction (Benjamini & Hochberg, 1995) was used to adjust *p* values for multiple comparisons to limit the probability of even one false discovery.

## 3 RESULTS

### 3.1 Detection of *Cryptococcus* spp. by culture

Cryptococcal CFUs were observed after culture on niger seed agar in 14/23 (61%) tree hollow samples studied (Table 2). *URA5*‐RFLP analysis identified all isolates as *C. gattii* VGI. Tree species in which cryptococcal CFUs were observed include: *A. floribunda*, *E. albens*, *E. camaldulensis*, *E. pilularis*, *E. robusta*, *E. tereticornis*, and *Melaleuca* spp. (Table 2).

### 3.2 Detection of *Cryptococcus* spp. using NGS technology

**Figure 1:**
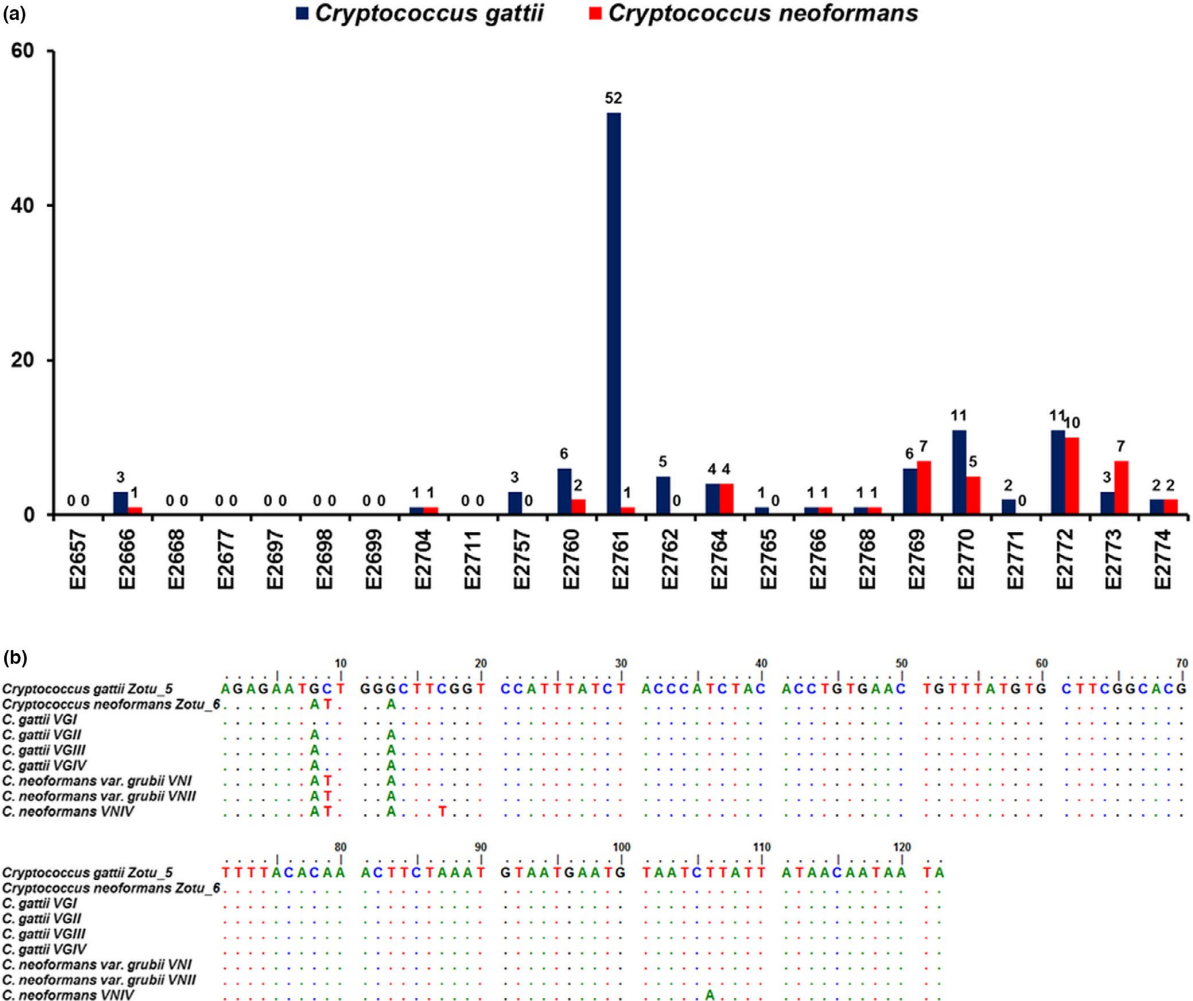
(a) Number of unique *Cryptococcus gattii*/*C. neoformans* species complexes ITS1 sequence reads detected from 23 tree hollows in New South Wales, Australia. (b) Alignment of the *C. gattii*/*C. neoformans* species complexes ITS1 region identified in next‐generation sequencing (Zotu_5 and Zotu_6) and the reference sequences of the *C. gattii*/*C. neoformans* species complexes (Katsu et al., 2004)

The bioinformatics tools were able to identify *C. gattii* or *C. neoformans* species complex specific reads in 16/23 (70%) samples. *C. gattii* species complex reads were detected in 16 samples, of which 12 were also positive for *C. neoformans* species complex reads. No *Cryptococcus* spp. reads were detected from seven samples (E2657, E2668, E2677, E2697, E2698, E2699, and E2711) (Figure 1a). In none of the samples were *C. neoformans* species complex reads recorded in the absence of *C. gattii* species complex reads. All identified *C. gattii* species complex reads were attributed to VGI, while all *C. neoformans* species complex reads were attributed to VNI/VNII. The hybrids between VNI and VNII, and interspecies hybrids, cannot be differentiated using high‐throughput amplicon sequencing.

Concordance between NGS and culture results was observed in 15/23 (65%) samples: 11 samples were simultaneously NGS‐ and culture‐positive for the *C. gattii* species complex while four samples were NGS‐ and culture‐negative for all *Cryptococcus* spp. In three samples (E2657, E2697 and E2699), *C. gattii* VGI was identified by culture but NGS failed to identify any *C. gattii* species complex specific reads. In another five samples, *Cryptococcus* spp. was identified using NGS, but culture‐based methods yielded negative results.

### 3.3 Mycobiome of eucalypt tree hollows

**Figure 2:**
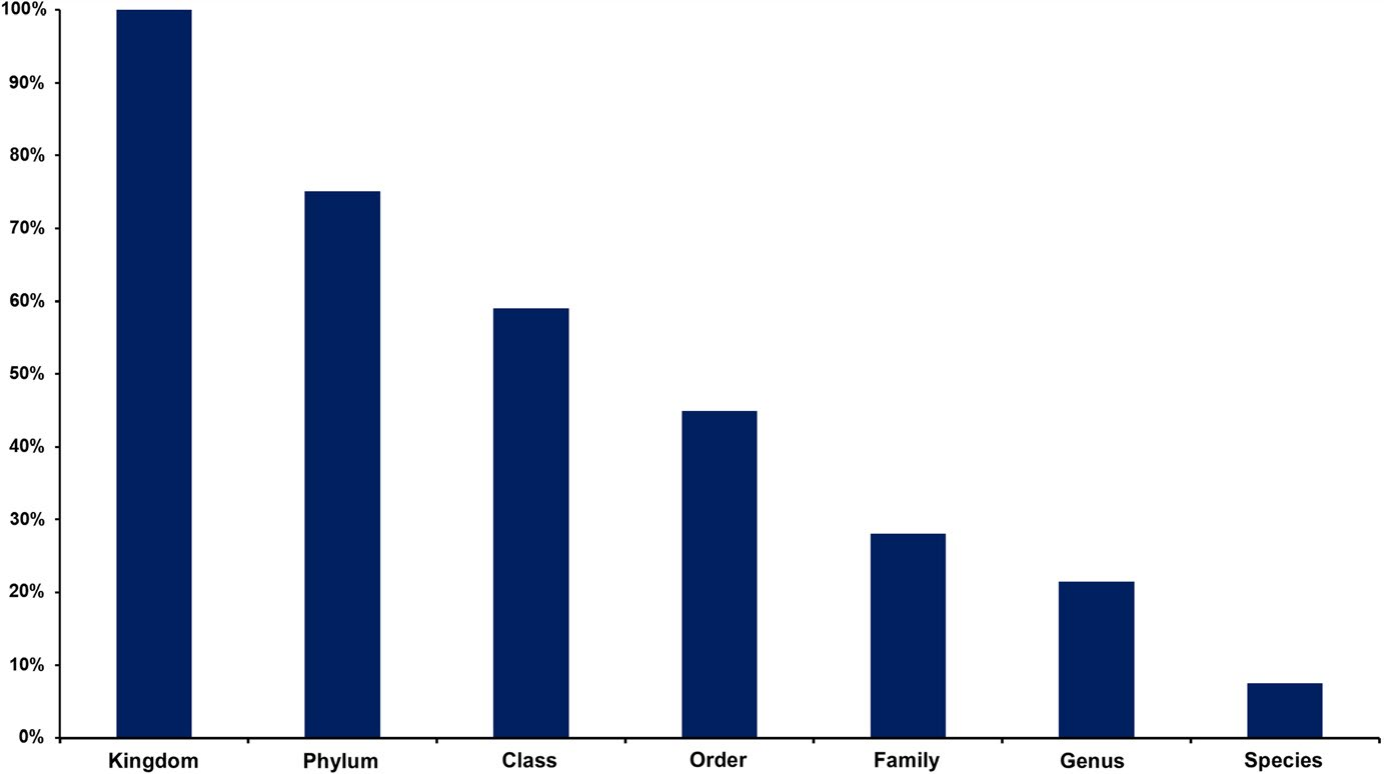
Operational taxonomic unit assignment rates at different taxonomical levels using ITS1 amplicon‐based metagenomics analysis of samples from 23 tree hollows in New South Wales, Australia

The number of generated sequence reads per sample was highly variable (mean 126,381; standard deviation [*SD*] 44,207) and thus the OTU table was normalized to the same number of reads (5,000) per sample. We identified a total of 2,638 OTUs that were assigned at different taxonomic levels: 199 to species (7.5%), 565 to genus (21.4%), 741 to family (28.1%), 1,185 to order (44.9%), 1,557 to class (59.0%), 1,982 to phylum (75.1%), and 2,638 OTUs to kingdom level (100%) (Figure 2). The number of OTUs was different in each sample, with the average of 550 and *SD* of 273.

**Figure 3:**
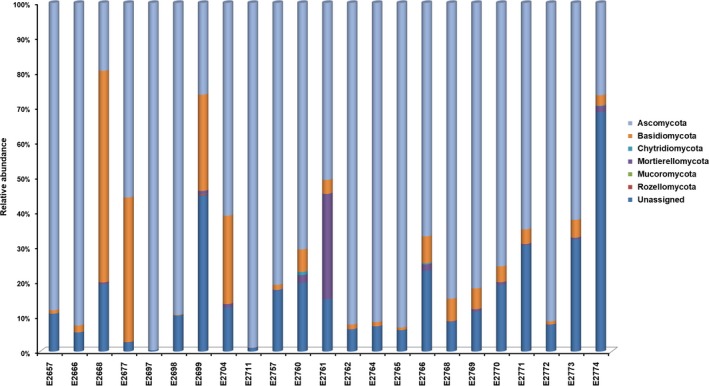
The inferred taxonomic composition of fungal communities at phylum level for environmental samples collected from 23 tree hollows in New South Wales, Australia

**Figure 4:**
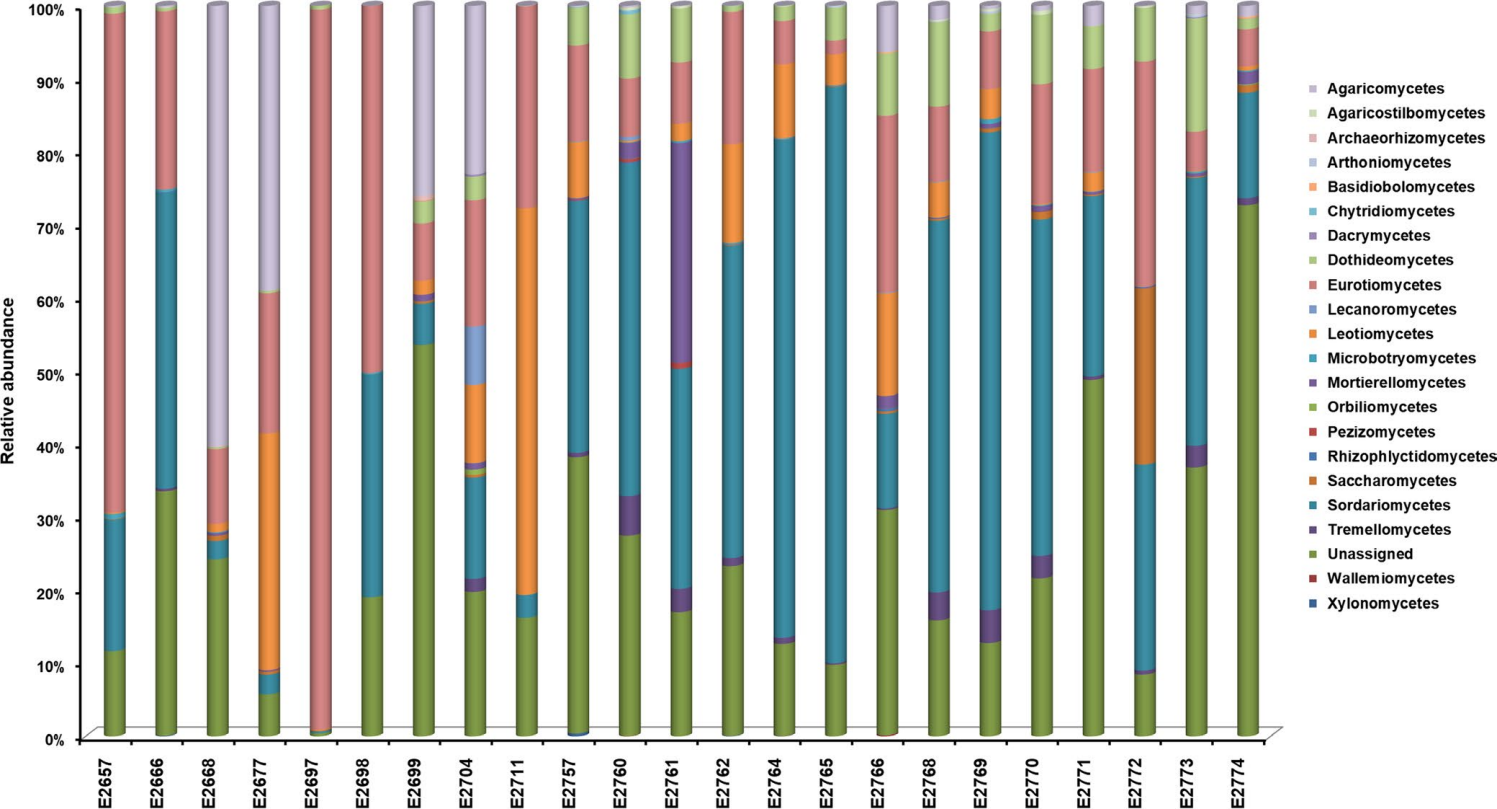
The inferred taxonomic composition of fungal communities at class level for environmental samples collected from 23 tree hollows in New South Wales, Australia

**Figure 5:**
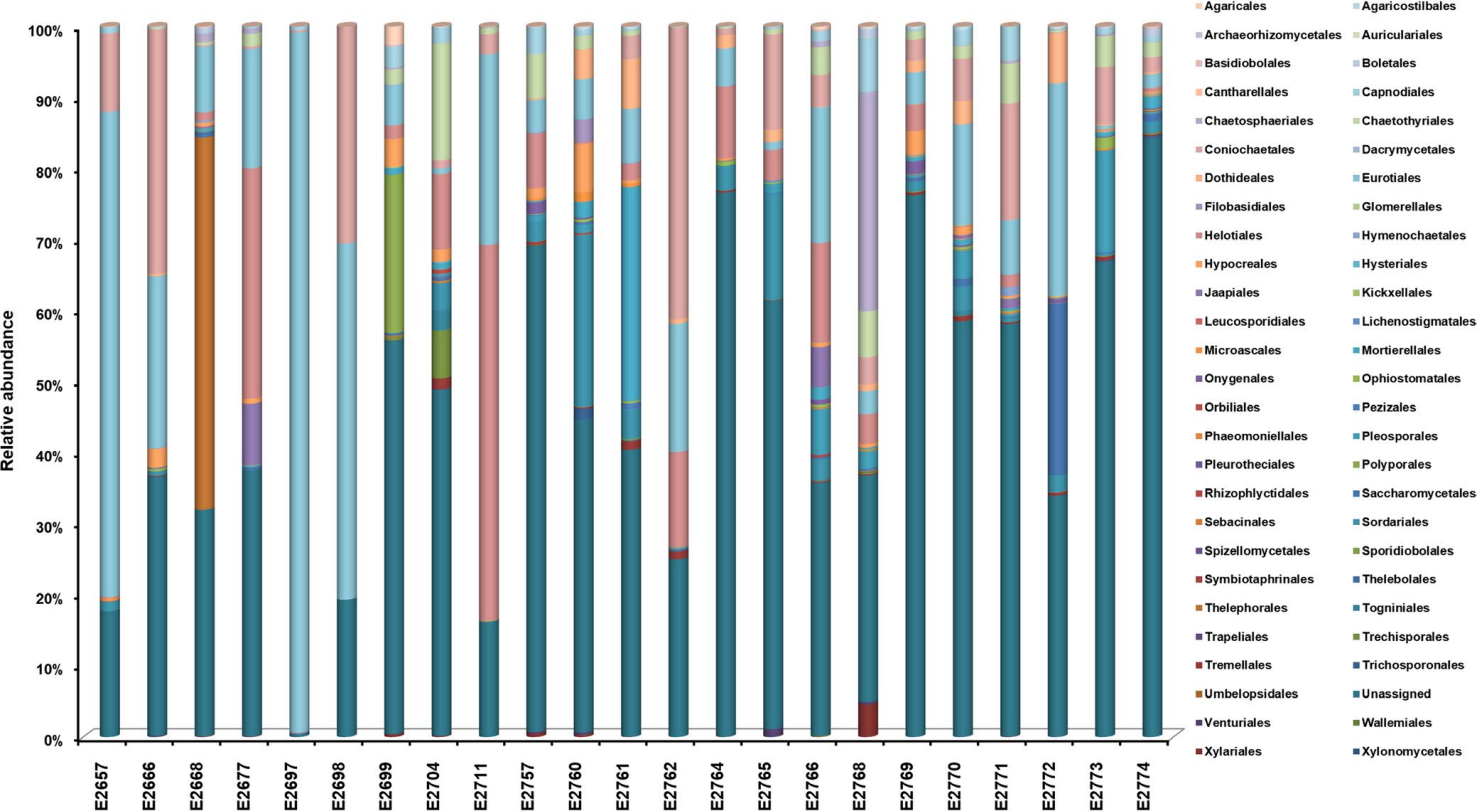
The inferred taxonomic composition of fungal communities at order level for environmental samples collected from 23 tree hollows in New South Wales, Australia

OTUs classified in the Ascomycota were observed most frequently (1,660 OTUs; 62.9% sequences), followed by the Basidiomycota (226 OTUs; 8.6% sequences; Figure 3). The distributions of taxa at class and order per sample are shown in Figures 4 and 5. Much less abundant fungi included Mortierellomycota (38 OTUs; 1.4% sequences), Chytridiomycota (22 OTUs; 0.8% sequences), Rozellomycota (18 OTUs; 0.7% sequences), and Mucormycota (12 OTUs; 0.5% sequences). Some 25% of the sequences (662 OTUs) remained unassigned at phylum level (Figure 2).

Among the Ascomycota, the classes with higher sequence abundance belonged to Sordariomycetes (28.1%), Eurotiomycetes (25.9%), Dothideomycetes (12.5%) Leotiomycetes (4.4%), and Saccharomycetes (2.7%). Among the Basidiomycota, the class Agaricomycetes was the most abundant (51.3% sequences) followed by Tremellomycetes (22.6%) and Microbotryomycetes (4.9%).

**Figure 6:**
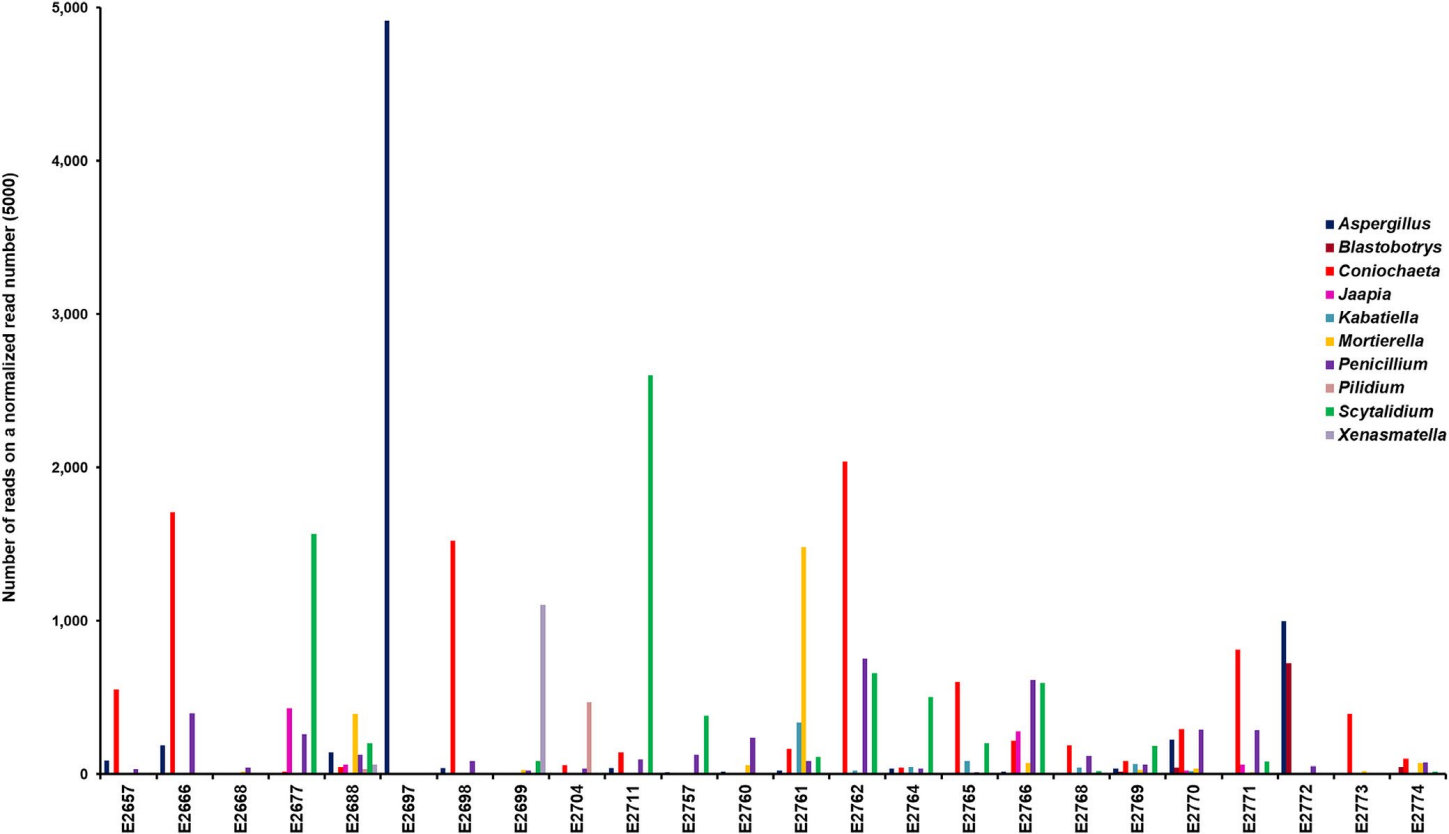
The 10 most abundant genera within the mycobiome of the 23 tree hollows in New South Wales, Australia, without the unassigned operational taxonomic unit on a normalized read number (5,000) for each sample

At genus level, among Ascomycota, *Penicillium* (2.3%), *Aspergillus* (1.6%), *Scytalidium* (1.5%), and *Coniochaeta* (1.3%) were the most abundant genera. In the Basidiomycota, *Trechispora* (4%), *Jaapia* (2.7%), and *Cryptococcus* (2.5%) were the most dominant. In Mortierellomycota, the most common genera were *Mortierella* (55.3%) and *Gamsiella* (2.6%). The overall ten most abundant genera in each sample are shown in Figure 6.

**Figure 7:**
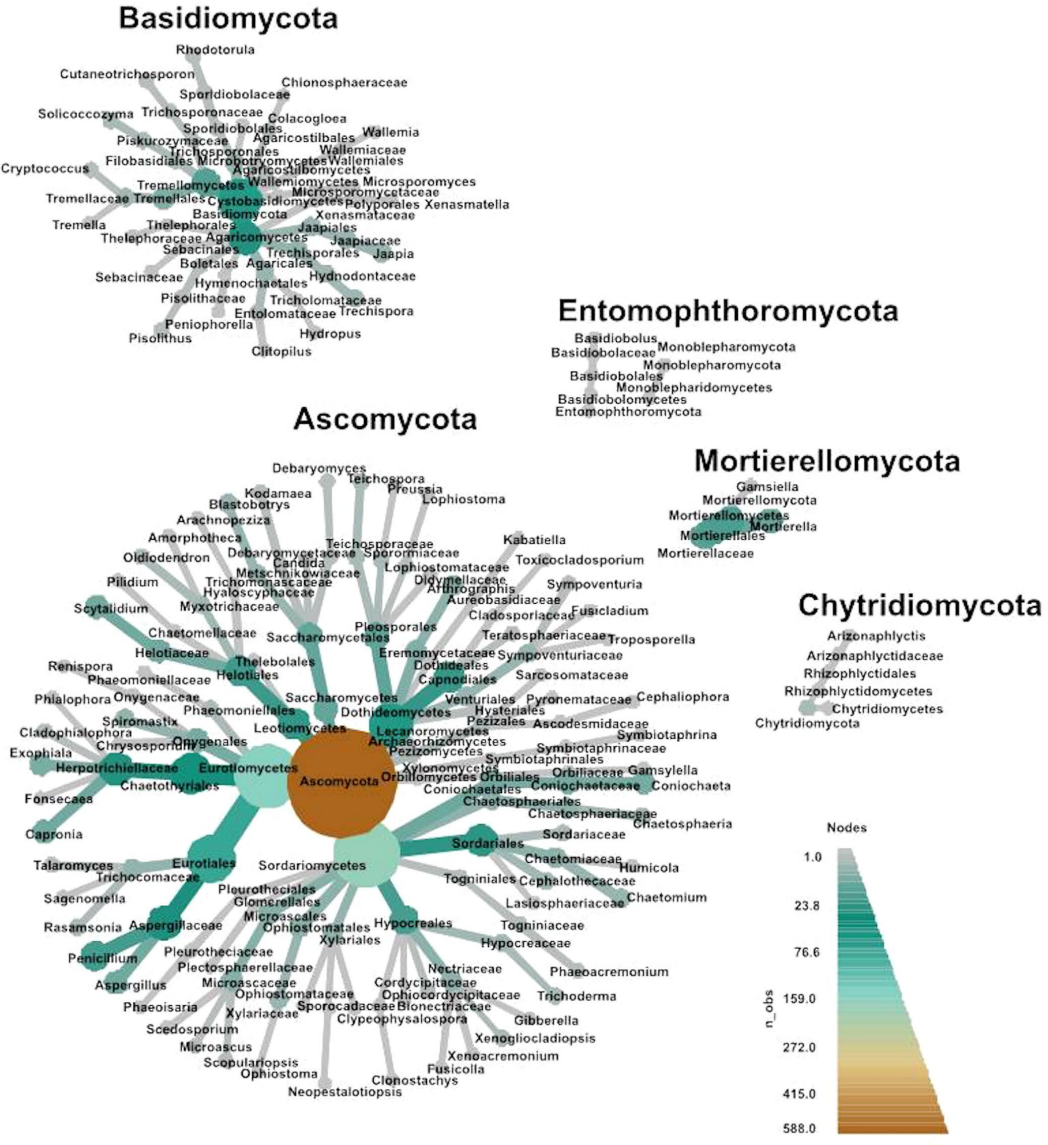
Heat tree of the abundance of taxa at different ranks of the 23 tree hollows in New South Wales, Australia. The size and color of nodes and edges are correlated with the abundance of taxa. The central node is the total of all the other nodes in the tree for each phylum

At species level, 901 OTUs (34.2%) were singletons, 371 (14.1%) and 205 (7.8%) OTUs were represented by only two and three reads, respectively. A total of 1,719/2,638 OTUs (65.2%) were represented by less than five reads in the samples. The most abundant genus among all samples was *Coniochaeta* (anamorph: *Lecythophora*), with *Aspergillus* and *Penicillium* the next most abundant genera. The overall abundance of taxa at different ranks of the 23 tree hollows are displayed in the heat tree (Figure 7).

**Figure 8:**
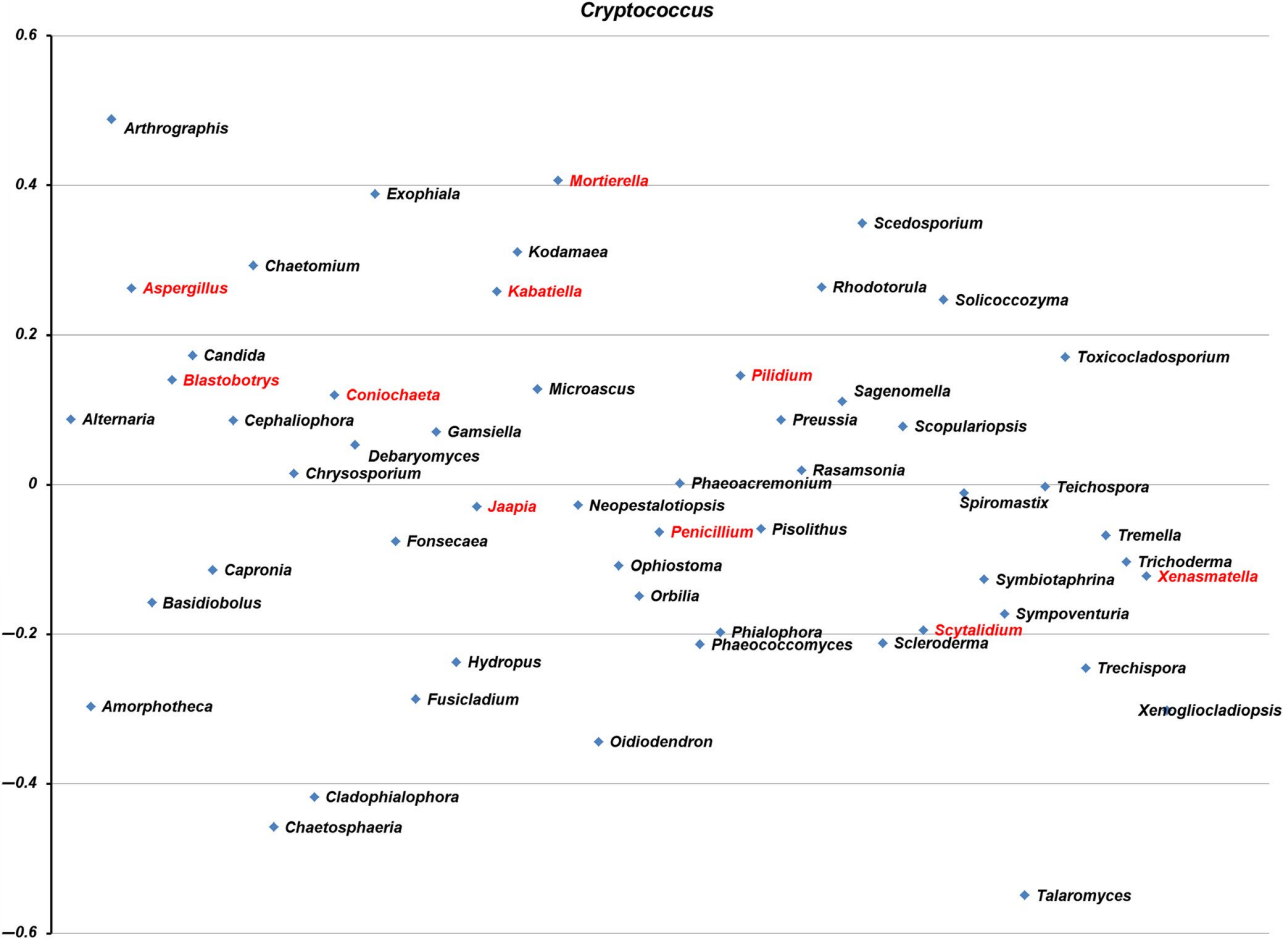
Correlation analysis using SparCC, Pearson, and Spearman correlations between *Cryptococcus* spp. and other fungal genera detected in the 23 tree hollows in New South Wales, Australia. Red text denotes the 10 most common genera. Genera are arranged alphabetically from left to right

Co‐occurrence analysis did not show any significant correlation between the occurrence of *Cryptococcus* spp. and other taxa or genera (Figure 8). The correlation analysis likewise did not show any statistical significance (*p* < 0.05) or negative or positive correlation between *Cryptococcus* spp. and other genera (Figure 8).

**Figure 9:**
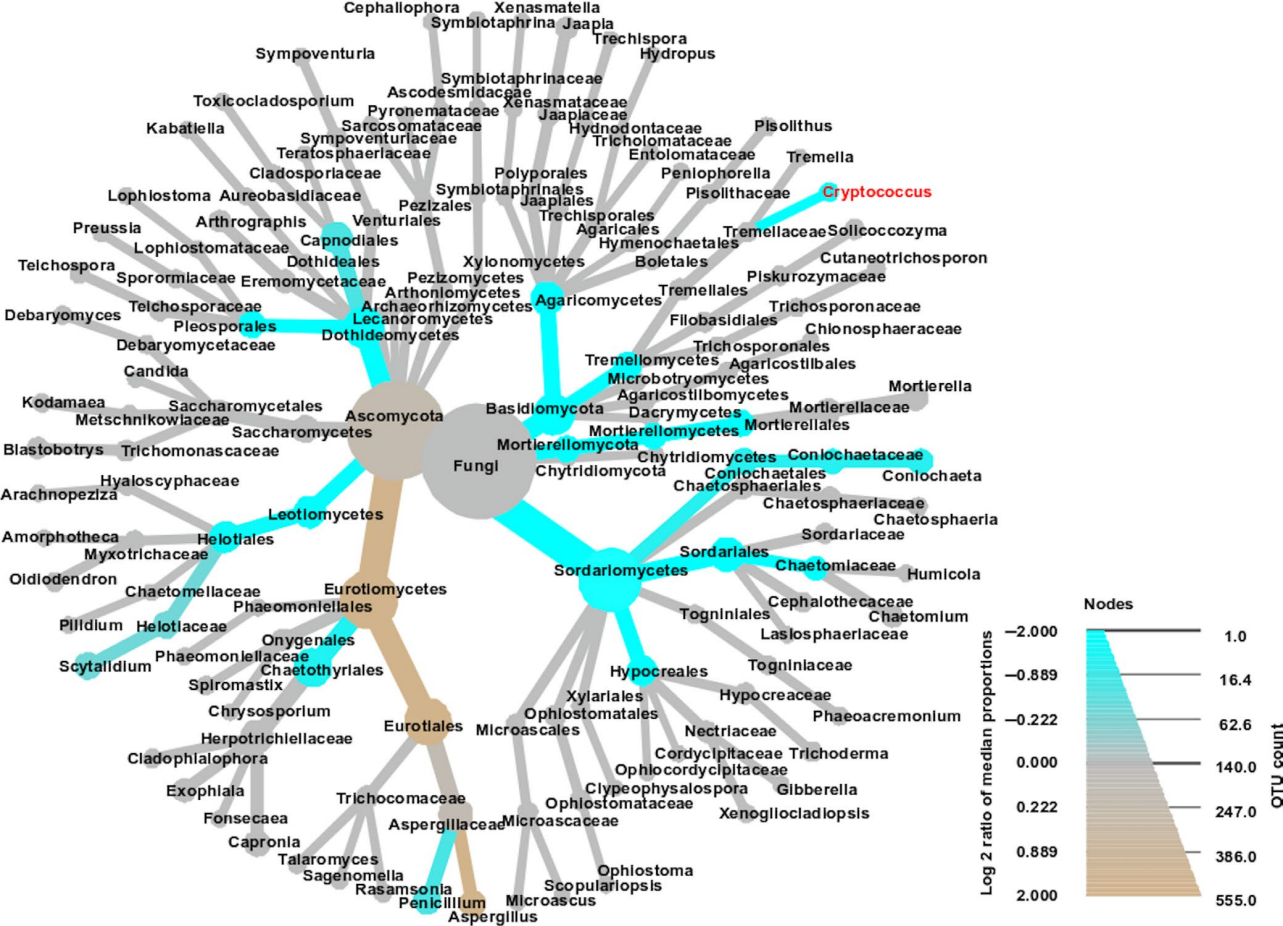
Taxa abundance tree with differential heat mapping in the presence of *Cryptococcus gattii/C. neoformans* species complexes in the mycobiome of 23 Australian tree hollows. The color of each taxon represents the log 2 ratio of median proportions of reads observed in *C. gattii/C. neoformans* species complex negative and positive samples. Taxa colored brown are more abundant in negative and those colored blue are more abundant in positive samples. Only significant differences are colored, as determined using a Wilcoxon rank‐sum test followed by a Benjamini‐Hochberg (FDR) correction for multiple comparisons

The differential heat tree analysis (Figure 9) highlighted numerous taxa that differed in the abundance between trees with no *Cryptococcus* spp. reads compared with those with one or more reads. In trees with no *Cryptococcus* spp. reads, Ascomycota, Eurotiomycetes, Eurotiales, and *Aspergillus* were comparatively more abundant at phylum, class, order, and genus level, respectively. In trees with *Cryptococcus* spp. reads, numerous taxa were identified as comparatively more abundant at phylum, class, order, and family levels. At the genus level, four genera were highlighted as being of greater abundance—*Coniochaeta*, *Cryptococcus*, *Penicillium*, and *Scytalidium* (Figures 6 and 9).

Diversity indices did not at all highlight any specific predictors likely to explain the occurrence of *Cryptococcus* spp. in certain tree hollows. Fungal communities varied, based on OTU numbers and Shannon and Evenness indices (Table 3). The number of detected OTUs in the sample varied between 125 and 1,062, with the average of 570. The Simpson index, which indicates whether a single large OTU dominates the sample, was also highly variable (0.022–0.952). The values closer to 1 indicated that a few OTUs dominated the sample contrary to a value closer to 0 indicating that the samples were composed of multiple OTUs, none of which dominated the sample. The Berger‐Parker and Robbins frequency indexes showed that the samples with more OTUs detected had many singletons. One particular sample (E2697) had Simpson and Berger‐Parker values indicative of a single large OTU dominating the sample. Figures 3, 4, 5, 6 confirm this pattern and indicate that the single dominant OTU in this sample can be attributed to *Aspergillus*.

## 4 DISCUSSION

In this study, we used amplicon‐based metagenomic analyses as a tool to characterize the mycobiome of 23 Australian tree hollows as a key environmental niche for *Cryptococcus* spp., the causal agent for both clinical and subclinical cryptococcosis in koalas. The other aim of this study was to detect *Cryptococcus* spp. and assess the correlation between the presence of *Cryptococcus* spp. in a tree hollow and the presence of any other fungal genus.

We characterized the mycobiome of Australian native tree hollows and found no definitive correlations between the presence of *Cryptococcus* spp. and other genera. Discrepancies were established between culture‐based and amplicon‐based metabarcoding methods for the detection of the *C. gattii* and *C. neoformans* species complexes in environmental samples. Although culture‐based methods and NGS were often in agreement, discordant results were observed in 8/23 samples. These findings can be explained by potential biases in both methods, which will be discussed. As a result of this study, the known environmental niche of *C. gattii* VGI was expanded by its detection in the hollows of another five tree species as follows: three eucalypts (*E. pilularis*, *E. populnea*, and *E. robusta*), a *Melaleuca* spp., and an *Angophora* spp.

Culture‐based identification remains challenging due to the rapid growth of other environmental fungi negatively impacting the ability of *Cryptococcus* spp. to grow in some samples. All conventional culture methods have limitations, such as the unknown and potentially low sensitivity, cost (use of multiple culture media), and turnaround time (often up to a week, potentially longer when the time required for subculturing and molecular confirmation of the phenotypic identification is considered). The slow‐growing nature of *Cryptococcus* spp. compared with many filamentous fungi can also cause delays. Given the unknown sensitivity of this method, perhaps repeated cultures of each sample in this study could have yielded different results.

It is also possible that cryptococcal DNA was detected using NGS but insufficient viable organisms were present in these samples for culture‐based detection to be successful. This observation may have clinical relevance and should be considered carefully if NGS results are used as part of a disease investigation, as viable live yeast cells or basidiospores are required to initiate an infection. The potential for environmental DNA to complicate NGS results is further explained later in the discussion. The culture‐positive but NGS‐negative results for *Cryptococcus* spp. are likely related to the multitude of biases encountered in amplicon‐based metagenomics, as discussed later. It is also of note that the number of *Cryptococcus* spp. reads based on NGS did not appear to be consistent with the culture‐based grading of low, moderate or heavy. These findings again suggest that abundance‐based results are generally considered unreliable in amplicon‐based metagenomics studies (Nguyen, Smith, Peay, & Kennedy, 2015; Tessler et al., 2017).

Many samples were found to contain *C. neoformans* VNI/VNII sequences using NGS, as this method is unable to distinguish between VNI and VNII since their ITS1 regions are identical (Figure 1b; Katsu et al., 2004). These findings were not supported by the culture results, as none of the isolates collected were identified as being members of the *C. neoformans* species complex using *URA5*‐RFLP analysis. This could be due to the potential high error rate of NGS and a very high similarity between the ITS1 regions of the *C. gattii* and *C. neoformans* species complexes. Differentiating between them relies on only three polymorphic sites in the ITS1 region (Figure 1b; Katsu et al., 2004), which may also explain that *C. neoformans* VNI/VNII reads were only obtained in samples that also had *C. gattii* VGI reads. However, the number of *C. neoformans* VNI/VNII reads suggested that they are unlikely to be solely attributable to sequencing error. It is also possible that members of the *C. neoformans* species complex were present in the samples but were in a quiescent state and therefore did not grow on the culture media (Hommel et al., 2019). The *Cryptococcus* reads after denoising were extracted, visually checked and their taxonomic assignments were confirmed using BLAST and pairwise alignment against the reference ITS sequences. The choice of another target, more discriminatory between the *C. gattii* and *C. neoformans* species complexes, such as the entire ITS region or the *URA5* gene (Meyer et al., 2003) may have circumvented this, but the size of both regions precluded their compatibility with the technology (MiSeq^®^ System of Illumina) used in this study. The use of long‐read sequencing technologies, such as MinION™ from Oxford Nanopore Technologies (Eisenstein, 2012), may improve discriminatory power by allowing for the sequencing of the entire ITS region or other targets, such as the *URA5* gene.

The mycobiome of Australian tree hollows characterized in this study appears to be reasonably consistent with our expectations of a decaying, hardwood microenvironment. Three genera: *Coniochaeta*, *Aspergillus*, and *Penicillium*, dominated the mycobiome. Species in the *Coniochaeta* genus (pleomorphic yeasts) are known pathogens of trees and are often isolated from necrotic wood samples but can also cause opportunistic human infections after traumatic implantation, such as keratitis, subcutaneous abscesses, peritonitis, and endocarditis (Damm, Fourie, & Crous, 2010; de Hoog, Guarro, Gené, & Figueras, 2000; Khan et al., 2013; Taniguchi et al., 2009). There is some speculation that yeasts may be overrepresented in NGS analyses due to their higher nucleus to cytoplasm ratio when compared to filamentous fungal species with longer cells (Lindahl et al., 2013). *Aspergillus* and *Penicillium* spp. are well‐known fungal genera associated with wood degradation (soft rot fungi) in nature, since they tolerate wide ranges of temperature, humidity, and pH, and attack a variety of wood substrates. Soft rot fungi are more common in hardwood, such as *Eucalyptus* spp., than in softwood which might be due to differences in the quality of the lignin (Hamed, 2013). Other genera, such as *Scytalidium, Blastobotrys, Jaapia*, or *Mortierella*, are also commonly found in decaying organic matter and produce enzymes that enhance the degradation of proteins in the wood of dead trees (Middelhoven & Kurtzman, 2007; Takahashi & Oda, 2008; Telleria, Dueñas, Melo, Salcedo, & Martín, 2015; Wagner et al., 2013). Statistical comparisons between tree hollow mycobiomes were not considered relevant in this study, because the primary focus of the sampling was to investigate the connection between cryptococcosis cases of koalas and the environmental source of these infections. A more in‐depth statistical analysis, requiring the systematic collection of further samples and additional information about trees and tree hollow characteristics will be subject of future studies. Further work of interest to the authors will be directed toward characterizing the mycobiome of the koala nasal cavity to determine how closely this reflects that of nearby tree hollows.

Although we identified 2,638 OTUs among the samples, only 7.5% were classified to species and 25.6% to genus level. Moreover, 34.2% of the OTUs were singletons which are largely due to the choice of the algorithm in the downstream analysis. Below order level, most OTUs remained unclassified without any taxonomic predictions. Our findings regarding the assignment success rate of OTUs agree with previous metagenomics studies carried out in a different environment (Schmidt et al., 2013; Soliman, Yang, Yamazaki, & Jenke‐Kodama, 2017; Sun et al., 2015; Yuan et al., 2017).

The lack of a relationship between the presence of *Cryptococcus* and any other fungal genera based on the correlation analysis is consistent with a prior study that also found no significant relationships between *Cryptococcus* and any other fungal taxa in environmental samples (Vanhove et al., 2017). However, the differential heat tree analysis suggested some differences in the relative abundance of numerous taxa in samples with versus without *Cryptococcus* reads. At genus level, *Aspergillus* was more abundant in *Cryptococcus*‐NGS‐negative samples and *Coniochaeta*, *Penicillium*, and *Sctyalidium* were more abundant in *Cryptococcus*‐NGS‐positive samples. However, both the correlation and heat tree analysis rely on NGS data, and as we have already demonstrated, NGS results may not always reflect the biological reality of the microbiome/mycobiome. Of particular note in this study is one tree in which a heavy growth of *C. gattii* VGI was observed on culture, yet no *Cryptococcus* reads could be identified using NGS (Table 2, E2657). As previously mentioned, abundance‐based results are also often considered unreliable in NGS studies (Nguyen, Smith, et al., 2015; Tessler et al., 2017). Therefore, it is difficult to draw definitive conclusions from these findings, and further work, such as more systematic sampling and numerous technical replicates would be required to determine how reliable these potential associations are. We also did not attempt to find correlations between *Cryptococcus* and bacteria or nonfungal eukaryotes in Australian tree hollows, which most likely will also influence the composition of the mycobiome but are beyond the scope of the current work.

The aforementioned NGS metabarcoding results should be interpreted with care, as a number of technical issues and biases inherent in amplicon‐based metabarcoding have been reported, including: PCR primer selection (Elbrecht & Leese, 2015; Pinto & Raskin, 2012), tag switching (Esling, Lejzerowicz, & Pawlowski, 2015; Schnell, Bohmann, & Gilbert, 2015), template concentration, amplification conditions (Kennedy, Hall, Lynch, Moreno‐Hagelsieb, & Neufeld, 2014), and PCR and sequencing errors (Nakamura et al., 2011; Tremblay et al., 2015; Turnbaugh et al., 2010). Such errors are difficult to distinguish from true biological variation (Edgar, 2016b; Leray & Knowlton, 2017). The denoising algorithm used in this study aims to remove sequencing noise and preserve all biological reads in the sample, an important step in NGS data analysis. We used the UNOISE improved error correction algorithm (Edgar, 2016b). However, denoising algorithms present a challenge and another potential bias of NGS data interpretation, as defining an abundance threshold that differentiates correct sequences from random errors is difficult (Schirmer et al., 2015). The high number of singletons observed in the dataset might have been attributable to the settings in UNOISE in the analysis. It is also well recognized that inferences from metagenomics studies are greatly influenced and varied by the fields, laboratory and analytic techniques utilized (Majaneva, Hyytiäinen, Varvio, Nagai, & Blomster, 2015). Besides technical biases in sample preparation, DNA extraction and sequencing, there is also another level of complexity and biases in downstream analyses and databases.

Another major limitation of NGS metabarcoding is the lack of reference databases, which are necessary to determine the phylogenetic affiliation of sequence reads. Taxonomical assignments are only as good as the reference databases (Santamaria et al., 2012). This study used the UNITE full dataset (Kõljalg et al., 2013) which also includes the ISHAM‐ITS (Irinyi et al., 2015) dataset containing all relevant ITS sequences of *Cryptococcus* spp. The SINTAX taxonomy classifier (Edgar, 2016a) was chosen to predict the taxonomy of the identified sequences, as it achieved comparable or better accuracy than the popular RDP Naive Bayesian Classifier (NBC) (Wang, Garrity, Tiedje, & Cole, 2007).

Another bias of amplicon‐based metagenomics is the uncertainty as to whether the detected DNA belongs to a live or a dead microbe. DNA is ubiquitous and stable in the environment and can account for roughly 10% of extractable phosphorus in soil (Turner & Newman, 2005). Extracellular DNA fragments can persist over time in many environments, allowing for their detection with high‐throughput sequencing technology (Nielsen, Johnsen, Bensasson, & Daffonchio, 2007). Eucalypt hollows could protect environmental DNA from some forms of degradation, including extreme heat and UV‐radiation.

Another interesting finding of this study is its presentation of the first published detection of the *C. gattii* species complex in Australia from *A. floribunda*, *E. pilularis*, *E. populnea*, *E. robusta*, and *Melaleuca* spp. to the best of our knowledge. The detection from *E. populnea* was, however, only based on NGS and could not be confirmed by culture. It is possible that this represents a sampling error when plated on culture media for the detection of fungal growth, nonviable organisms or erroneous reads due to the inherent biases associated with NGS metabarcoding. Nevertheless, it is becoming increasingly clear that the ecological niche of *C. gattii* VGI in Australia extends far beyond the classic association with *E. camaldulensis* and *E. tereticornis*.

Based on our findings of discrepancies between culture and NGS‐based identification of *Cryptococcus* spp. from environmental samples, neither approach can be considered definitive. Spiking samples with *Cryptococcus* spp. could prove a useful future experiment to further assess this. NGS potentially could not differentiate between the *C. gattii* and *C. neoformans* species complexes, no significant correlation between the presence of *Cryptococcus* spp. and other fungal genera or taxa could be identified, and abundance‐based analyses were inconclusive. As expected, the mycobiome of Australian tree hollows reflected the microenvironment of decaying wood. Given the discrepancies between culture and NGS results and the multitude of potential biases in amplicon‐based metagenomics, meaningful inferences are difficult to establish, and results must be interpreted with caution. Further improvements in NGS, such as whole‐genome shotgun and long‐read sequencing, together with appropriate data analysis pipelines and the extension of reference databases should significantly contribute to better characterization and understanding of such complex microbial community structures. Further work in this area should include assessing possible correlations between *Cryptococcus* spp. and bacteria, free living ameba, and nematodes.

## AUTHOR CONTRIBUTIONS

L.J.S., L.I., R.M., W.M., and M.B.K. designed research; L.J.S. and L.I. performed research; L.J.S., L.I., and J.R.P. analyzed data; and L.J.S., L.I., R.M., J.R.P, W.M., and M.B.K. wrote the manuscript.

## Data Availability

All raw reads from this study were submitted to NCBI's Sequence Read Archive under the BioProject accession PRJNA497337.
